# Injectable Phage-Loaded Microparticles Effectively Release Phages to Kill Methicillin-Resistant *Staphylococcus aureus*

**DOI:** 10.1021/acsami.3c19443

**Published:** 2024-03-30

**Authors:** Yajing Xu, Tao Yang, Yao Miao, Qinglei Zhang, Mingying Yang, Chuanbin Mao

**Affiliations:** † School of Materials Science and Engineering, Zhejiang University, Hangzhou 310058, Zhejiang, China; ‡ Institute of Applied Bioresource Research, College of Animal Science, Zhejiang University, Yuhangtang Road 866, Hangzhou 310058, Zhejiang, China; § Department of Biomedical Engineering, The Chinese University of Hong Kong, Shatin 999077, Hong Kong SAR, China

**Keywords:** bacteriophage, microparticle, drug-resistance, antibacterial agents, abdominal infection

## Abstract

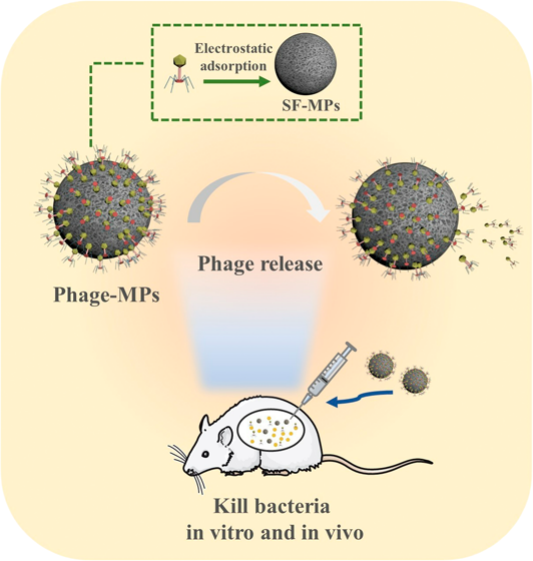

The increasing prevalence of bacterial multidrug antibiotic resistance has led to a serious threat to public health, emphasizing the urgent need for alternative antibacterial therapeutics. Lytic phages, a class of viruses that selectively infect and kill bacteria, offer promising potential as alternatives to antibiotics. However, injectable carriers with a desired release profile remain to be developed to deliver them to infection sites. To address this challenge, phage-loaded microparticles (Phage-MPs) have been developed to deliver phages to the infection site and release phages for an optimal therapeutic effect. The Phage-MPs are synthesized by allowing phages to be electrostatically attached onto the porous polyethylenimine-modified silk fibroin microparticles (SF-MPs). The high specific surface area of SF-MPs allows them to efficiently load phages, reaching about 1.25 × 10^10^ pfu per mg of microparticles. The Phage-MPs could release phages in a controlled manner to achieve potent antibacterial activity against methicillin-resistant *Staphylococcus aureus* (MRSA). Unlike the diffuse biodistribution of free phages post-intraperitoneal injection, Phage-MPs could continuously release phages to effectively boost the local phage concentration at the bacterial infection site after they are intraperitoneally injected into an abdominal MRSA-infected mouse model. In a mouse abdominal MRSA infection model, Phage-MPs significantly reduce the bacterial load in major organs, achieving an efficient therapeutic effect. Furthermore, Phage-MPs demonstrate outstanding biocompatibility both in vitro and in vivo. Overall, our research lays the foundation for a new generation of phage-based therapies to combat antibiotic-resistant bacterial infections.

## 1 Introduction

Bacterial infections pose a significant public health threat worldwide, leading to a high number of illnesses and deaths.^1,2^ The discovery of penicillin in the 20th century initially controlled numerous infectious diseases.^3^ However, widespread, irrational antibiotic use accelerated clinically significant bacterial resistance within a few years, resulting in tens of thousands of global deaths annually.^2,4^ Despite continuous efforts to develop new antimicrobials, the pace significantly lags behind rising demand and escalating bacterial resistance. Since the 1980s, only three new antibiotics were discovered, highlighting a critical gap.^5−8^ A 2017 report from the World Health Organization’s Global Antibiotic Surveillance System emphasizes the dire state of antibiotic resistance, with associated annual treatment costs reaching approximately $20 billion.^9,10^ It is predicated that there will be a potential annual death toll of 10 million by 2050 due to antibiotic-resistant pathogens.^11,12^ Without intervention, superbugs will persist, rendering current antibiotics ineffective in 10 to 20 years.^13^ While antibiotics have been the go-to treatment for bacterial infections,^14^ their efficacy is increasingly limited due to the rise of bacteria resistant to antibiotics, especially methicillin-resistant *Staphylococcus aureus* (MRSA).^15^ MRSA is widespread in hospitals and communities worldwide, exhibiting high morbidity and mortality rates.^16^ MRSA exhibits extremely high resistance, rendering the majority of β-lactam antibiotics ineffective.^17^ Vancomycin serves as the ultimate antibiotic, yet challenges persist, including issues related to toxicity and efficacy. Consequently, alternative treatment strategies such as lytic bacteriophages have gained considerable attention in recent years.^18,19^ Lytic bacteriophages (phages) are a type of virus that targets and destroys bacteria.^20^ These natural predators are abundant in nature and can be easily isolated and characterized, making them a promising alternative to antibiotics in combating bacterial infections.^21^ Unlike antibiotics, bacteriophages are highly specific to their target bacteria and do not harm human cells or beneficial bacteria in the body.^22,23^ Moreover, they can be modified to target antibiotic-resistant bacteria, making them a valuable alternative to antibiotics.^24^ Additionally, bacteriophages have a unique mechanism of action that involves replicating within the host bacteria, resulting in their lysis and death. This mechanism prevents the development of bacterial resistance because the bacteriophages can evolve and adapt to the bacterial strain.^25,26^

However, using bacteriophages as a therapy for bacterial infections faces several challenges.^27^ One of the most significant challenges is the efficient delivery of bacteriophages to the infection site.^28^ In addition, bacteriophages are sensitive to environmental conditions, such as pH and temperature, and can be easily degraded before reaching the site of infection.^22^ Therefore, an effective delivery system is essential for successfully applying bacteriophages in treating bacterial infections.^29^

**Figure 1:**
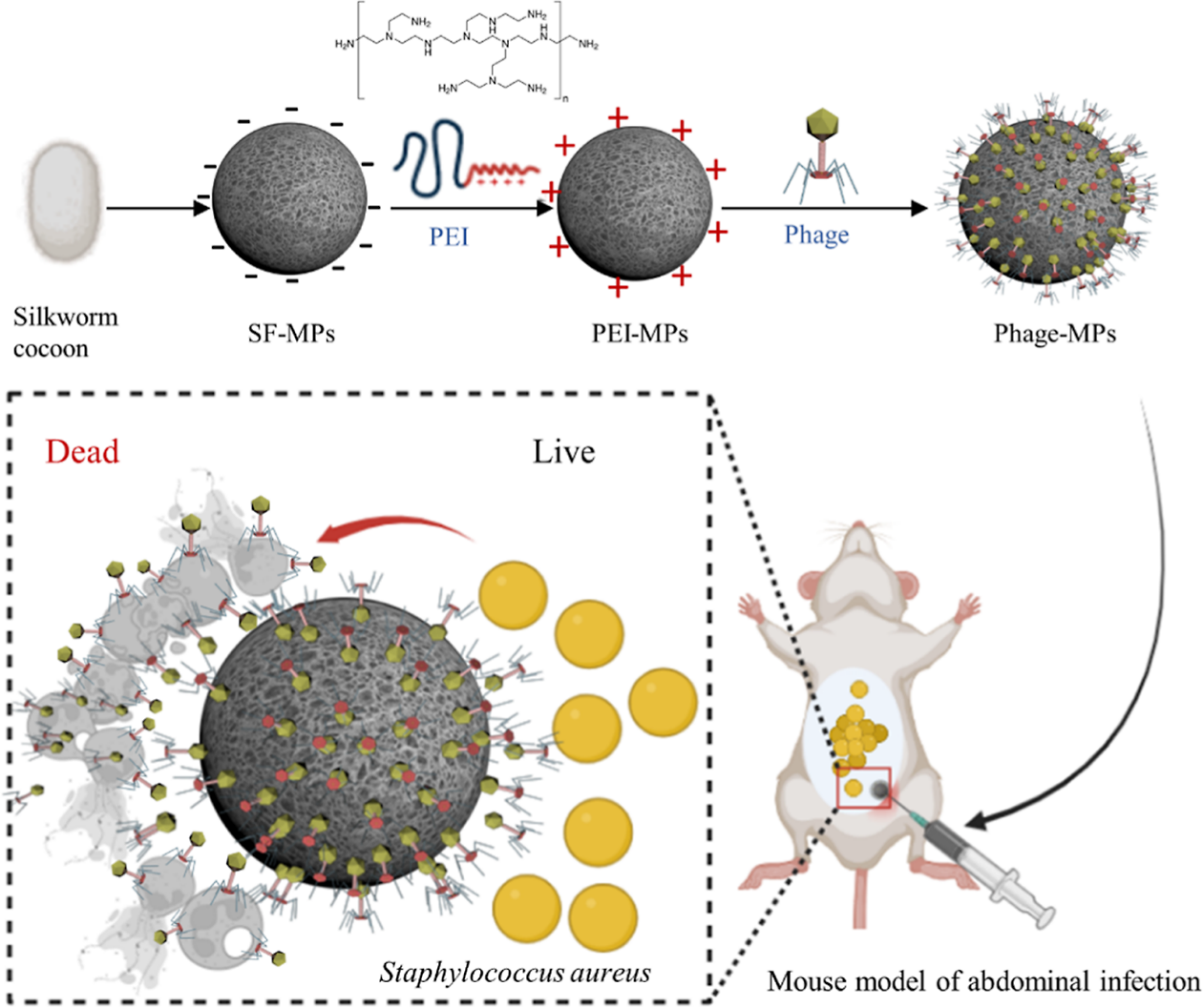
Schematic illustration of the development of phage-loaded microparticles (Phage-MPs) for inactivating bacteria and treating abdominal infection in the mice model. SF was first isolated from cocoons and then processed into microparticles (SF-MPs) through a microemulsion method. The negatively charged SF-MPs were further modified with positively charged polyethylenimine (PEI) to become PEI-MPs. The negatively charged phages were then electrostatically attached to PEI-MPs, generating Phage-MPs. The Phage-MPs were administered into mice with abdominal MRSA infection via intraperitoneal injection. They release phages to kill MRSA, effectively treating the MRSA infection.

Our group has found that silk fibroin (SF) can be developed into a drug delivery system.^30−32^ SF is a natural protein derived from silkworm cocoons and has several advantages over other carriers, such as synthetic polymers^33,34^ and liposomes.^35,36^ It is biocompatible, biodegradable, and can be processed into biomaterials of various sizes and shapes.^37^ Furthermore, the resultant biomaterials are expected to protect the loaded cargo from degradation and enhance its stability.^38^ Hence, we proposed assembling SF into microparticles (SF-MPs), then using SF-MPs to load bacteria-attacking phages to form phage-loaded MPs (Phage-MPs), and finally employing Phage-MPs to destroy MRSA both in vitro and in vivo. Specifically, we screened the phages that could specifically infect *S. aureus* from sewage and attached it to SF-MPs to form Phage-MPs by electrostatic adsorption (Figure 1). We found that the phages could be released from Phage-MPs in a controlled and sustained manner over time. Consequently, the released phages effectively killed MRSA in vitro and in vivo with no observable cytotoxicity, demonstrating the potential of Phage-MPs in the delivery of bacteriophages for treating clinically relevant bacterial infections.

## 2 Materials and Methods

### 2.1 Materials

Petroleum ether (PE), sodium carbonate (Na_2_CO_3_), anhydrous ethanol, sodium chloride (NaCl), and isopropyl alcohol were obtained from Sinopharm. Lithium bromide (LiBr) and Span-80 were purchased from Aladdin. Branched polyethylenimine (PEI, Mw ∼ 25,000) was acquired from Sigma-Aldrich. Polyethylene glycol 8000 (PEG-8000), vancomycin, yeast extract, tryptone, agar power, DNase I, phosphate-buffered saline (PBS), penicillin/streptomycin, and a CCK-8 assay kit (CA1210) were purchased from Solarbio. A live/dead staining kit (L34951) was obtained from Thermo Fisher Scientific. FITC and rhodamine B were purchased from Macklin.

### 2.2 Microbial Strains

The bacterial species utilized in this research were *S. aureus* 9118 and methicillin-resistant *S. aureus* N315 (MRSA) that were separated from human clinical specimens.

### 2.3 Cell Culture

The human epithelial 293T cells (Shanghai Institute of Cell Biology, Chinese Academy of Sciences) were cultured in DMEM containing 10% (v/v) FBS and 1% penicillin–streptomycin solution.

### 2.4 Synthesis of Silk Fibroin Microparticles

The process of extracting silk fibroin (SF) from cocoons was conducted following established protocols.^39^ Initially, the cocoons were fragmented into small pieces, immersed in a solution containing 0.02 mol/L of sodium carbonate, and boiled for 30 min, then washed and dried, redissolved in 9.3 mol/L lithium bromide solution, dialysis and concentrated, and kept in a 4 °C refrigerator for later use.

Silk fibroin microparticles (SF-MPs) were synthesized through microemulsion and freezing phase separation. First, 0.7 mL of Span-80 was mixed with 45 mL of PE. The mixture was stirred constantly to dissolve completely. Then, it was added with 5 mL of SF solution (7%) containing 0.5 mL of isopropyl alcohol, followed by stirring at room temperature for 1–2 min to obtain a W/O microemulsion. Subsequently, the microemulsion was quickly poured into the −40 °C precooled PE. The mixture was left to settle for 15 min to remove the organic solvent. The SF MPs were freeze-dried for 48 h and stored at −20 °C.

### 2.5 Scanning Electron Microscopy of MPs

A small amount of SF-MPs was placed onto a scanning electron stub, and gold sputtering was performed to apply a thin coating onto the sample. The images were captured by an SU-8010. The particle size was characterized by counting the scanning electron microscopy (SEM) images with Photoshop software.

### 2.6 Isolation and Purification of Phage

The phage samples were derived from natural sewage. *S. aureus* 9118 and MRSA were both used as host bacteria for phage screening. First, the sewage sample was subjected to centrifugation at 10,000 rpm for 15 min and filtered at 0.22 μm to remove impurities and bacteria. Then, the filtered sample (10 mL) was combined with a 2× LB medium (50 mL). After 200 μL of activated *S. aureus* was added, the suspension was cultured at 37 °C and 220 rpm overnight. The mixture was centrifuged for 15 min at 10,000 rpm. The resulting supernatant was filtered to obtain the phage stock solution, which was subsequently stored in a refrigerator (4 °C) for later use. The presence of phages in the obtained stock solution was carried out through a double agar overlay method.

The independent plaque was picked out on the plate and put into a microcentrifuge tube with 1 mL of PBS buffer. It was placed at 25 °C for 1 h and then kept at 4 °C for another 8 h. Then, the mixture was centrifuged for 10 min at 10,000 rpm to acquire a phage supernatant. 100 μL diluted phage and 100 μL activated *S. aureus* (OD_600_ = 0.3) were mixed and then purified by the double agar overlay method. They were repeated 3–4 times until the size of the plaque showed no more changes.

### 2.7 Amplification of Purified Phage

Phages were propagated in an LB liquid medium. Initially, the host bacteria were cultured until the prelogarithmic stage. Then, phages were added to the host bacteria culture, which was maintained at 37 °C with shaking at 220 rpm until lysis occurred. After lysis, DNase I was added at 0.1 μg/mL and the culture was further incubated 15 min at 37 °C. The culture was then centrifuged at 12,000 g for 20 min to eliminate bacterial debris. The phage solution was concentrated by precipitation with 1 mol/L NaCl and 10% PEG 8000, and the mixture was stored overnight at 4 °C. The culture was centrifuged again at 12,000 g for 20 min, and the supernatant was decanted. The resulting pellet was resuspended in PBS containing 10% PEG 8000, followed by centrifugation and resuspension in PBS. The purified phage solution was obtained by centrifuging the solution again and collecting the supernatant. The purified phage solution was stored at −80 °C in solutions containing 8% glycerol.

### 2.8 Phage Plaque Assay

The quantity of phages was confirmed by using the double-layer plate method. The LB agar plate with a single layer was prepared beforehand. Then, 100 μL of diluted phage solution and equal volume of *S. aureus* (OD_600_ = 0.3) were mixed, followed by coculturing at 37 °C for 15 min. Five milliliter top agarose was added, and it was promptly poured onto the prepared LB broth agar plate to create a second layer. When the top agarose hardened, the plate was inverted and incubated at 37 °C overnight. The phage titer (pfu, plaque-forming unit) then could be calculated by the number of plaques.

### 2.9 Transmission Electron Microscopy of Phage

A clean copper mesh was taken and coated with 5–10 μL of phage solution, then incubated for 30 min. Excess liquid was removed using filter paper, and the mesh was subsequently stained for 2 min with 2% PTA. After removing excess staining solution by filter paper, the mesh was left to dry. The morphology of the phage was then observed by a Tecnai G2 F20 S-TWIN.

### 2.10 Loading of Phage into MPs

The SF-MPs were suspended in a solution of 5% branched polyethylenimine (PEI), shaking for 4 h, forming a layer of PEI molecules on the surface of SF MPs. After washing three times with deionized water, 4 mg PEI-MPs were resuspended in 2 mL phage solution (∼10^11^ pfu/mL) and incubated for 4 h. The obtained Phage-MPs were then washed several times using PBS.

### 2.11 Quantification of Phage Loaded on MPs

The decreasing number of phage titers between the original solution and the remaining solution after adsorption onto the MPs was used to calculate the total phages attached to the surface of the MPs. The double agar overlay method was used to measure the phage quantity.

### 2.12 Fluorescent Staining of Phage-MPs

First, PEI was labeled with FITC. Specifically, a mixture of 500 mg of PEI, 4 mg of FITC, and 6.28 mg of DMAP was dissolved in a 10 mL of DMF solution. The solution was stirred at room temperature for 12 h, followed by a 2-day dialysis and subsequent storage at 4 °C for future use. Free phages were labeled with rhodamine B. The pH of the phage was adjusted to 8.0 using a 0.3 M NaHCO_3_ solution. Rhodamine B, dissolved in DMSO at a concentration of 1 mg/mL, was then mixed with the phage solution, incubated for 4 h, dialyzed for 2 days, and preserved at 4 °C. Finally, Phage-MPs were prepared using FITC-labeled PEI and Rhodamine B-labeled Phage by following the same steps as above.

### 2.13 Phage Release from MPs

Phage-MPs (4 mg) were added to 3 mL of PBS in a 6-well plate with mild shaking at 4 °C. At specific time points, 10 μL of incubation solution was taken out and replaced with 10 μL of fresh PBS. The phage release rate was calculated by the double-layer plate method.

### 2.14 In Vitro Antibacterial Activity

Antibacterial activity was assessed by the plate-count assay. Phage-MPs were suspended and diluted with a LB medium. 50 μL bacteria solution (OD_600_ = 0.3) was incubated with 500 μL different concentrations of Phage-MPs (0, 25, 50, 100, 200, and 400 μg/mL), ensuring a total volume of 2 mL. After being cultured at 37 °C for 3 h, a LB medium was used to dilute the bacterial solution 1000 times. Then, the diluted bacteria solution (100 μL) was evenly distributed onto the agar plates and cultured for another 12 h, followed by the observation of colonies. The bacterial counts were analyzed using ImageJ.

SEM was used to analyze the bacterial morphological changes under different conditions. Initially, the bacteria cells were immobilized using 2.5% glutaraldehyde and then dehydrated gradually using a sequence of ethanol solutions. Ultimately, the dried bacterial cells were visualized using SEM after they were coated with gold.

The antibacterial activity under various conditions was further examined by a live/dead bacterial staining assay. Specifically, the bacterial precipitate was obtained through centrifugation at 4000*g* for 5 min and washing with 0.85% NaCl solution. A 3 μL dye mixture consisting of equal propidium iodide and SYTO 9 was introduced to each mL of the bacterial suspension and incubated for 15 min. In addition, images were captured by inverted fluorescence microscopy. Bacterial cells that were still alive were stained green by SYTO 9 dye, whereas those with damaged cell walls were labeled red by a propidium iodide dye.

### 2.15 Hemolytic Activity Test

Fresh blood was obtained from mouse eyeballs and then centrifuged to isolate red blood cells (RBCs). After being washed five times with PBS, the RBCs were diluted to a 5% v/v concentration. Next, 500 μL of different groups of MPs (1 mg/mL) were added into 500 μL of the RBC solution. The mixture was kept in the 37 °C incubator for 6 h. The negative and positive controls were PBS buffer and 0.1% Triton X-100, separately. After centrifuging at 1000 g for 5 min, 100 μL supernatants were moved to a 96-well plate and OD_545_ were recorded. The value of hemolysis was analyzed using the following equation where A is the OD value of the sample, PBS, or Triton X-100
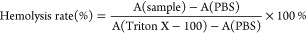


### 2.16 In Vitro Biocompatibility Study

To evaluate the biocompatibility of the MPs, the CCK-8 experiment was applied using the human epithelial 293T cell line.100 μL 293T cells were inoculated in 96-well plates (around 5000 cells a well) and incubated under humidified air containing 5% CO_2_ for 24 h at 37 °C. Next, 10 μL of different MP groups (1 mg/mL) were added to the 96-well plate. 293T cells treated with PBS served as a control. Then, the 96-well plate was cultured under humidified air containing 5% CO_2_ at 37 °C for another 24 h. Ten microliter of 10% CCK-8 solution diluted with DMEM was introduced into the well. Afterward, the plate was placed in the incubator for 1 h, and the OD_450_ was recorded via a multimodal microplate reader. The ratio of cell survival was analyzed using the following equation where A is the OD value for the sample, blank, or PBS
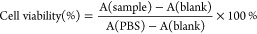


### 2.17 In Vivo Antibacterial Study

Male BALB/c mice (∼20g) were obtained from Shanghai SLAC Laboratory Animal Co., Ltd. The mice were arbitrarily assigned to 5 groups (n = 10) and fatally infected by injecting MRSA (500 μL, OD_600_ = 0.3) into the peritoneal cavity. After 1 h of inoculation, mice were administered with 1000 μL of the different MP groups (1 mg/mL), vancomycin (0.5 mg/mL) as a positive group, or PBS as a negative control. The body weight change and survival rate were recorded every 12 h. At 24, 48, and 72 h, three mice from the treatment group were euthanized randomly, and blood was collected from eyeballs. Meanwhile, the major organs were divided into two parts. One part was stained with H&E for histologic analysis, and another part was weighed and homogenized in cold PBS. The above solution was diluted 100 times with PBS, and 100 μL of the homogenized dilutions was evenly applied to the LB agar plates. The bacterial counts were analyzed using ImageJ software.

### 2.18 Statistical Analysis

All experiments were performed at least thrice. Statistical analysis was performed using GraphPad Prism 8. Results were presented as mean ± standard deviation (SD), and data was analyzed using one-way ANOVA and Tukey’s test. Asterisks suggest significant differences (*p < 0.05, **p < 0.01, ***p < 0.001).

## 3 Results and Discussion

### 3.1 Preparation and Characterization of Phage-MPs

**Figure 2:**
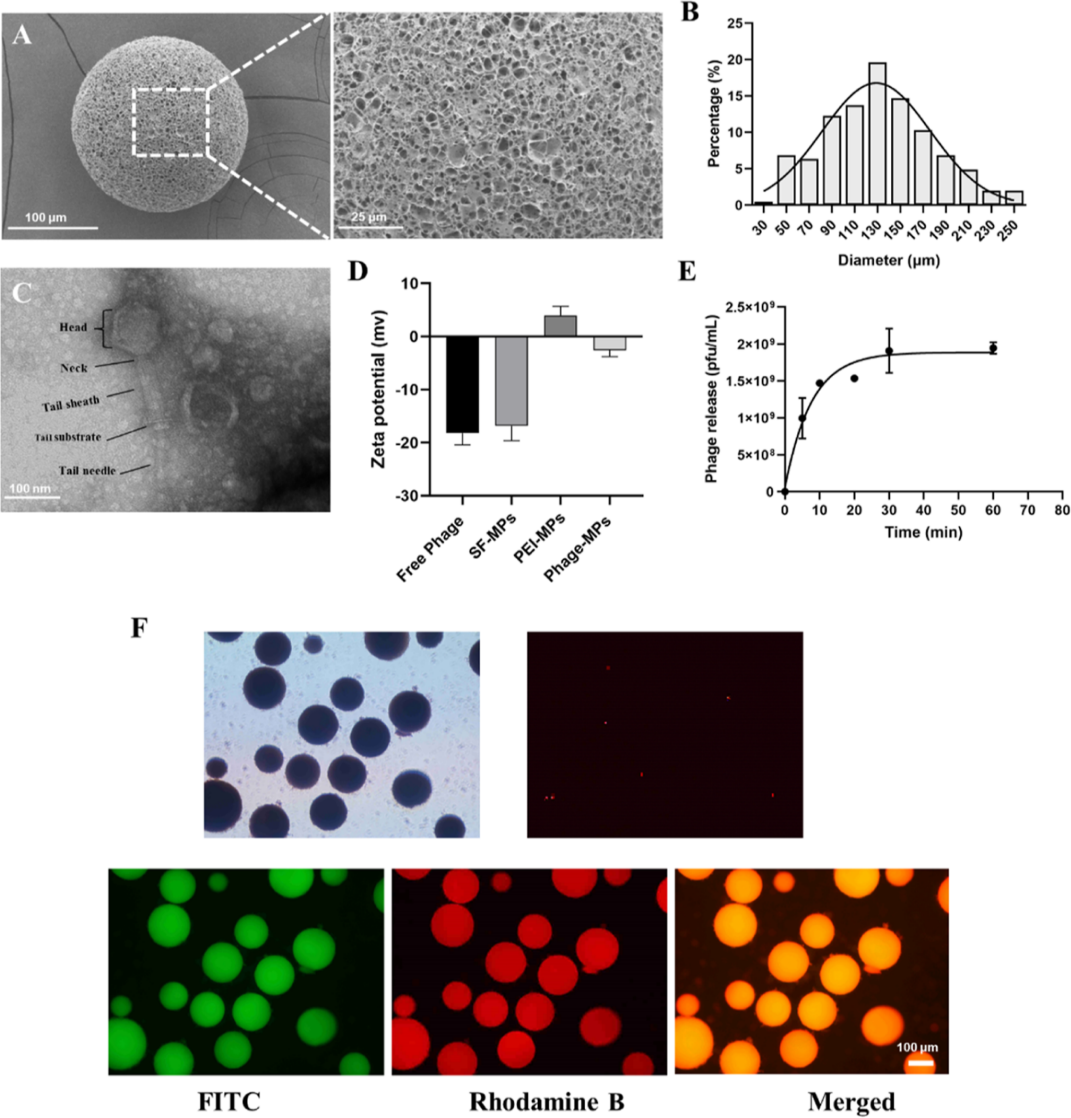
Characterization of Phage-MPs. (A) Representative SEM image of porous SF-MPs. (B) Size distribution of porous SF-MPs. (C) TEM image of phage isolated from sewage. (D) Zeta potentials of Free Phage, SF-MPs, PEI-MPs, and Phage-MPs. (E) Release of phages from Phage-MPs. Phage release was tested for 60 min after the microparticles were suspended in PBS. (F) Fluorescence images of Phage-MPs and the free phage. SF-MPs were labeled with FITC. Free Phage was stained with Rhodamine B.

SF-MPs were synthesized using the water-in-oil microemulsion method, followed by the removal of excess organic solvents by using a freezing phase separation technique. Briefly, three solutions, SF solution, span-80, and petroleum ether (PE), which served as the aqueous phase, surfactant, and oil phase, respectively, were mixed together to create a microemulsion. The microemulsion was then transformed into SF-MPs by rapidly freezing the liquid microparticles using a −40 °C precooled PE solution, followed by freeze-drying (Figure S1 in the Supporting Information). By adjusting the preparation parameters, we obtained highly porous and uniform spherical SF-MPs with a uniform particle size distribution (Figure S2 in the Supporting Information). The SEM image showed that the resulting SF-MPs with a size of about 132 μm were in a porous fibrous structure with a large specific surface area and could carry a large number of phages (Figure 2A, B).

A specific lytic phage against *S. aureus* was screened from sewage (Figure S3 in the Supporting Information). The transmission electron microscopy (TEM) image clearly revealed the structure of the phage, including the head, neck, tail substrate, tail sheath, and tail needle, with an icosahedral head measuring about 90 nm in diameter and a retractable tail around 200 nm in length (Figure 2C). When the isolated phage infected *S. aureus*, a bright plaque could be seen on the double agar plate, proving that the obtained phage can successfully lyse and kill *S. aureus* (Figure S4 in the Supporting Information).

The zeta potentials of SF-MPs and free phage were both negative, about −16.8 mV and −18.2 mV, respectively (Figure 2D). To facilitate the electrostatic adsorption of phages onto SF-MPs, polyethylenimine (PEI) with a branched structure and a molecular weight of 25 kDa was chosen based on its commercial availability and acceptable cytocompatibility. The zeta potential increased to 4.0 mV when SF-MPs were coated with a layer of PEI (termed PEI-MPs), enabling the negatively charged phages to be electrostatically bound to the PEI-MPs, as evidenced by a decrease in the surface potential to −2.6 mV upon phage attachment. These results confirm the successful decoration of SF-MPs with phages to generate Phage-MPs. The successful loading of phages onto SF-MPs was further demonstrated through fluorescent staining. PEI and phages were individually labeled with FITC and Rhodamine B. The two fluorescence signals completely overlapped (Figure 2F), confirming the electrostatic adsorption of phages onto SF-MPs. Because the porous SF-MPs offer a large surface area for phage adsorption, the amount of the loaded phages also increases with the increase of the phage input. When the phage input was 10^11^ pfu, the amount of the loaded phages was about 1.25 × 10^10^ pfu per mg of MPs (Figure S5 in the Supporting Information). Subsequently, we investigated the release kinetics of phages from Phage-MPs. Phages were released rapidly in the initial 30 min, followed by slow and sustained release (Figure 2E). This release pattern is ideal; it allows for a burst of phages to kill bacteria rapidly upon infection, and the subsequent gradual release prevents latent bacterial infection and maintains biocompatibility under physiological conditions. When free phages were utilized, the swift immune clearance diminished their presence, and their dispersion throughout the body hindered the achievement of an effective concentration at the infection site.^40,41^

### 3.2 In Vitro Antibacterial Activity

**Figure 3:**
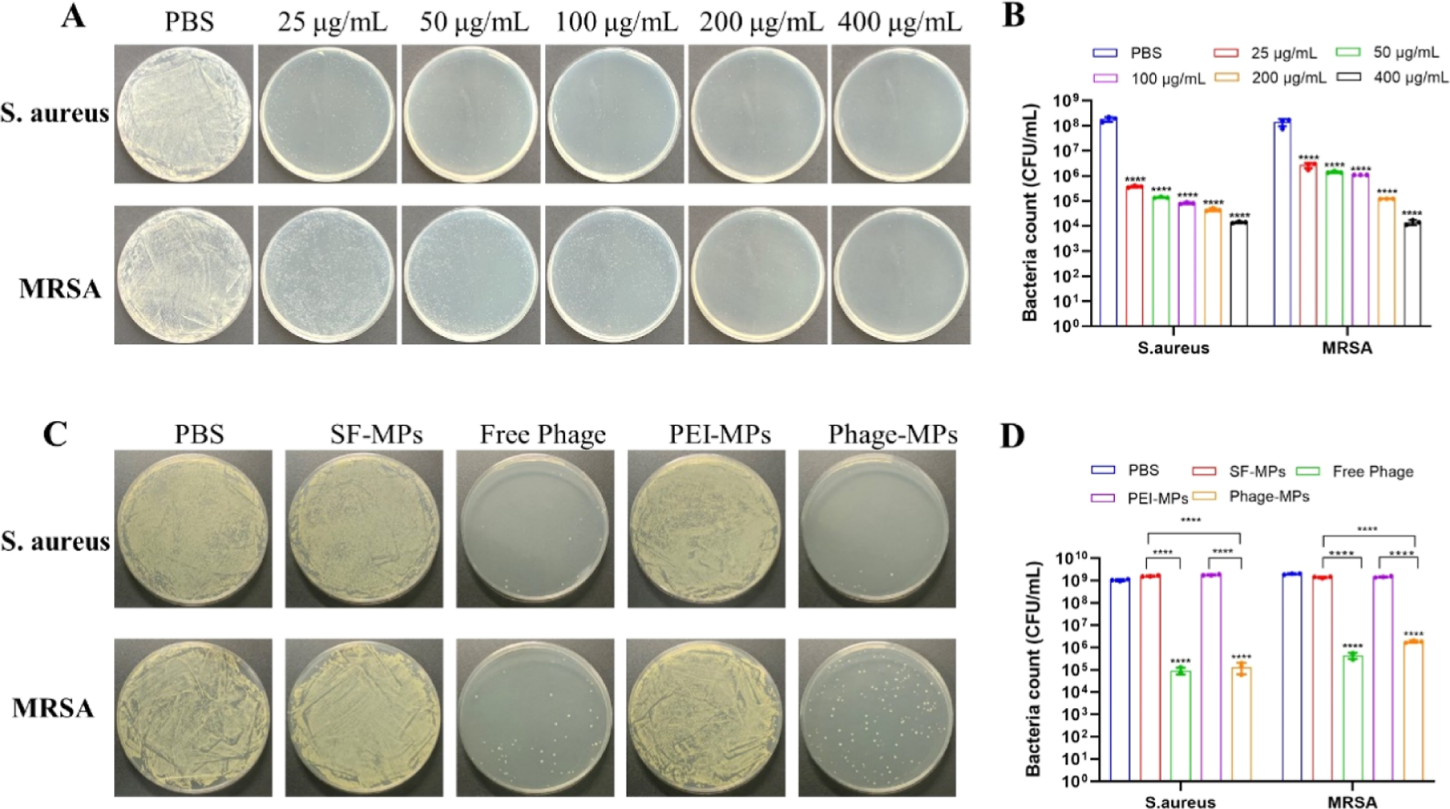
Antibacterial activity of Phage-MPs. (A) Photographs of bacterial colonies formed after the bacteria were treated by Phage-MPs at different concentrations. (B) Quantitative assessment of bacteria counts after treatment of Phage-MPs at different concentrations. (C) Photographs of bacterial colonies after the bacteria were treated by different groups. (D) Quantitative assessment of bacteria counts after treatment of different groups.

Phages are highly specific in their mode of action and can infect and replicate only within their host bacteria, making them a promising alternative to broad-spectrum antibiotics. Phages are also able to evolve and adapt to changes in the bacterial host, allowing for continued efficacy against resistant strains. To assess the potential of Phage-MPs as a substitute for antibiotics, *S. aureus* and MRSA were selected to investigate the in vitro antibacterial activity and their potential as a therapeutic agent against antibiotic-resistant bacteria. As shown in Figure 3A, the Phage-MPs had a remarkable antibacterial effect both against *S. aureus* and MRSA at very low concentrations compared to the PBS group. At a concentration of 25 μg/mL, the antibacterial rates of *S. aureus* and MRSA were 99.7% and 98.1%, respectively. The antibacterial effect was further strengthened as the concentration increases. The antibacterial effects of Phage-MPs with different concentrations were also quantitatively shown in Figure 3B. Although the bactericidal efficiency of the Phage-MPs against MRSA was slightly lower than that of *S. aureus* in the concentration range tested in the experiment, the bactericidal efficiency of both reached 99.9% at a bacteria concentration of 400 μg/mL, with almost no bacterial colonies visible on the plate. Unless otherwise specified, 400 μg/mL was used in all subsequent experiments.

Next, we studied the antibacterial activity of different groups (Figure 3C, D). Neither SF-MPs nor PEI-MPs had a significant antibacterial effect compared with the PBS group. Meanwhile, few colonies were seen on the agar plate in the Free Phage and Phage-MPs groups, which demonstrated that the bactericidal activity of the Phage-MPs was comparable to that of the Free Phage group. Importantly, the titer of active phages was comparable between those of the Phage-MP group and the Free Phage group, indicating that the electrostatic adsorption of phages onto the MPs did not significantly impact the bactericidal efficacy of the phages. These results validate the potential of electrostatically adsorbed phages onto MPs as a promising strategy for developing effective and safe phage-based antibacterial therapies.

**Figure 4:**
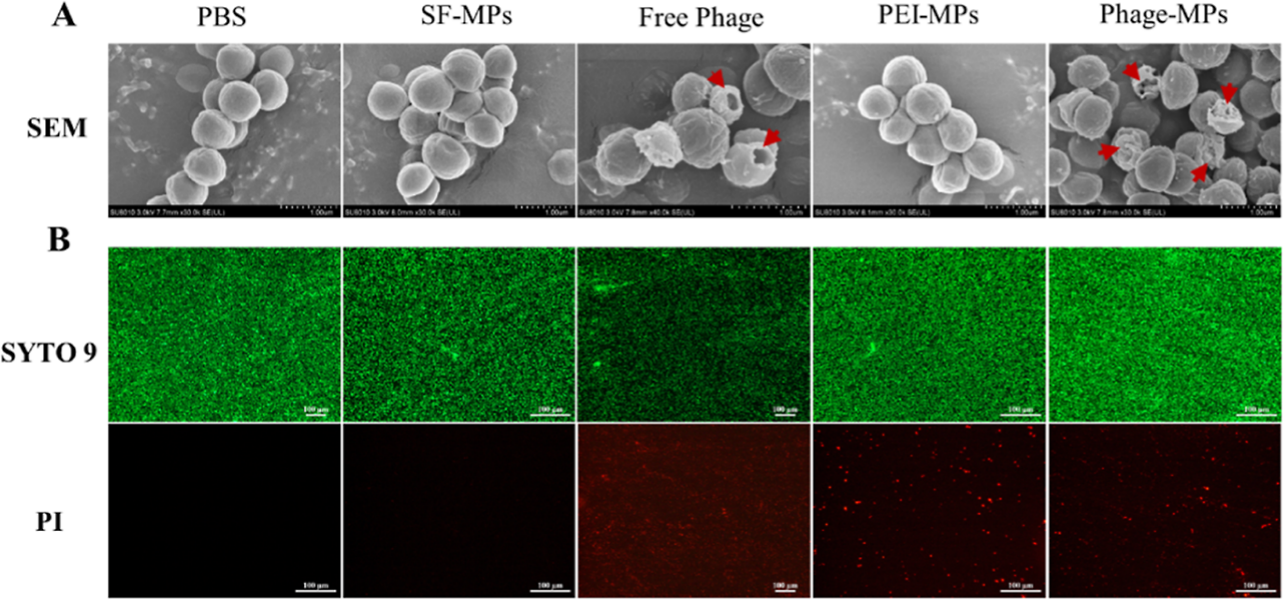
Killing of MRSA by Phage-MPs. Photographs of (A) SEM images and (B) live–dead staining of MRSA after different treatments. SYTO 9-stained viable cells (green), whereas dead cells were stained with PI (red). The red arrows indicate deep holes formed due to the attack of bacteria by the phage.

In order to better understand the antibacterial results described above, the morphological changes and viability of MRSA were further investigated by SEM observation and a LIVE/DEAD staining assay. As shown in Figure 4A, the untreated MRSA exhibited a contact morphology with a smooth surface. Following interactions with SF-MPs and PEI-MPs, only minimal damage on the surface of the bacteria was observed, indicating that the empty MPs (i.e., without phages) were relatively nontoxic to MRSA. However, the exposure to the Free Phage and Phage-MP groups resulted in a significant collapse of the cell envelope of MRSA, and deep holes appeared (red arrows in Figure 4A), demonstrating the strong bactericidal effects of phages. In Figure 4B, the results of the bacterial viability assay were consistent with the morphological changes observed in Figure 4A. The untreated MRSA cells showed predominantly green fluorescence, indicating that they were mainly live. In contrast, the SF-MP and PEI-MP groups exhibited a similar fluorescence pattern to the PBS group, with a large number of live bacteria stained green and presenting almost no red signal. However, in the Free Phage and Phage-MP groups, a significant number of dead bacteria were observed with red fluorescence signals, indicating the disruption of bacterial cell membranes induced by the phages. The red fluorescent signal was attributed to the penetration of propidium iodide fluorescent dye into the bacterial cells with a damaged membrane. In particular, PEI-MPs had a positive charge attributed to the presence of the positive PEI layer, so they would damage the negatively charged cell membranes to a certain extent. Consequently, a minor presence of red fluorescence was also observed in the PEI-MPs group. These results revealed that the Phage-MPs could release phages as well as cause significant damage to the MRSA cell walls, leading to MRSA death, further confirming the potential effectiveness of Phage-MPs as a viable antibacterial treatment for MRSA infections.

### 3.3 Hemocompatibility and Cytotoxicity Study

**Figure 5:**
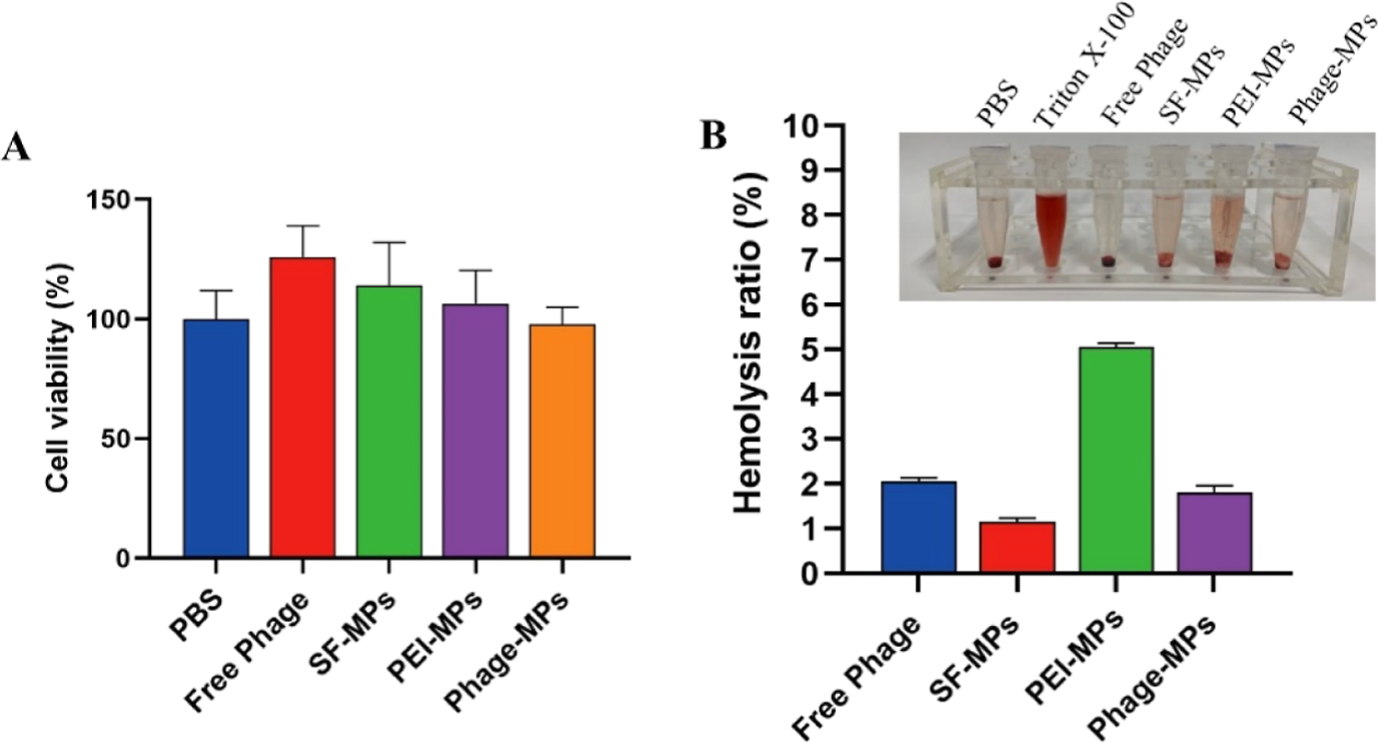
In vitro evaluation of biocompatibility. (A) Cell viabilities of 293T cells treated by PBS, Free Phage, SF-MPs, PEI-MPs, and Phage-MPs by the CCK-8 assay. (B) Hemolysis percentage and photographs (inset) of red blood cell solutions treated by different groups.

Now that the Phage-MPs were proven effective in releasing phages and exerting potent antibacterial activity against *S. aureus*, including the highly drug-resistant strain MRSA, we carried out cytotoxicity and hemolysis assays to investigate the biocompatibility of the microparticles in order to ensure the safety of using Phage-MPs as antibacterial agents. The cytotoxicity of the MPs was analyzed using 293T cells, and their viability was tested by the CCK-8 assay. There was no significant difference in cell viability between 293T cells treated with Phage-MPs and the PBS group, and all the groups had negligible cytotoxicity (Figure 5A). Next, a standard hemolysis assay was performed to evaluate the hemolytic potential, and the results were compared to the positive and negative controls (Figure 5B). The results showed that unlike Triton X-100, which fully lysed the red blood cells and released hemoglobin, no obvious hemoglobin release was observed after exposure to Free Phage, SF-MPs, and Phage-MPs. The hemolysis rate for PEI-MPs was slightly increased due to their positive charge, but it still remained below the permissible hemolytic level of 5.0%, indicating good hemocompatibility of the materials. The results revealed that the Phage-MPs exhibited minimal toxicity toward red blood cells and mammalian cells, indicating their acceptable cytocompatibility. These findings further ensured the potential of using Phage-MPs for killing bacteria, especially drug-resistant strains such as MRSA.

### 3.4 In Vivo Antibacterial Efficacy

**Figure 6:**
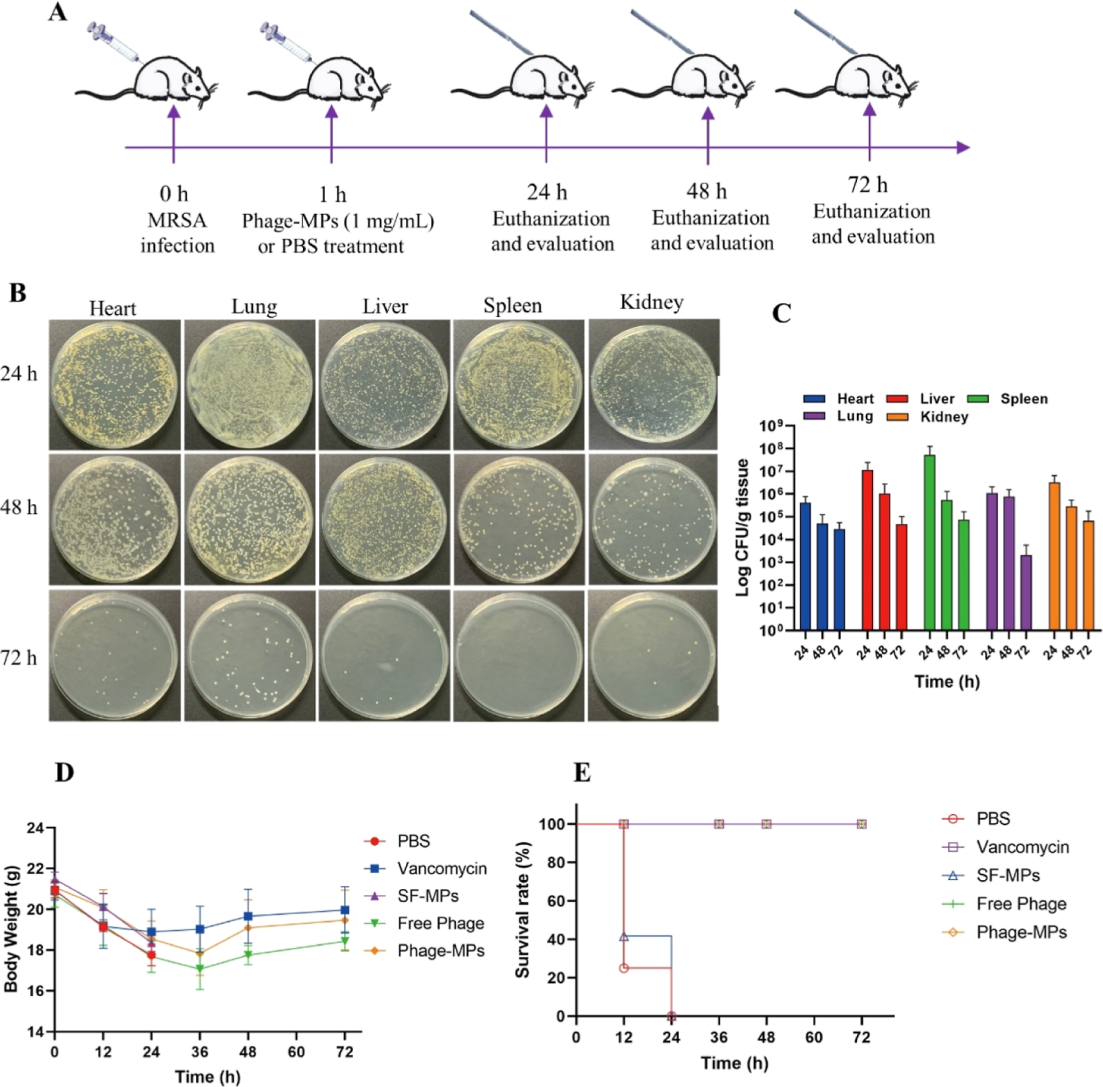
In vivo evaluation of the Phage-MPs on abdominal MRSA-infected mice. (A) Schematic of the experimental process of the mouse abdominal infected model. (B) Photographs of bacterial colonies of different tissue homogenates after treatment by Phage-MPs. (C) Quantitative assessment of MRSA counts in the heart, liver, spleen, lung, and kidney after the first infection with MRSA and then treatment with Phage-MPs. Body weight change (D) and survival rate (E) of MRSA infected mice after different treatments.

**Figure 7:**
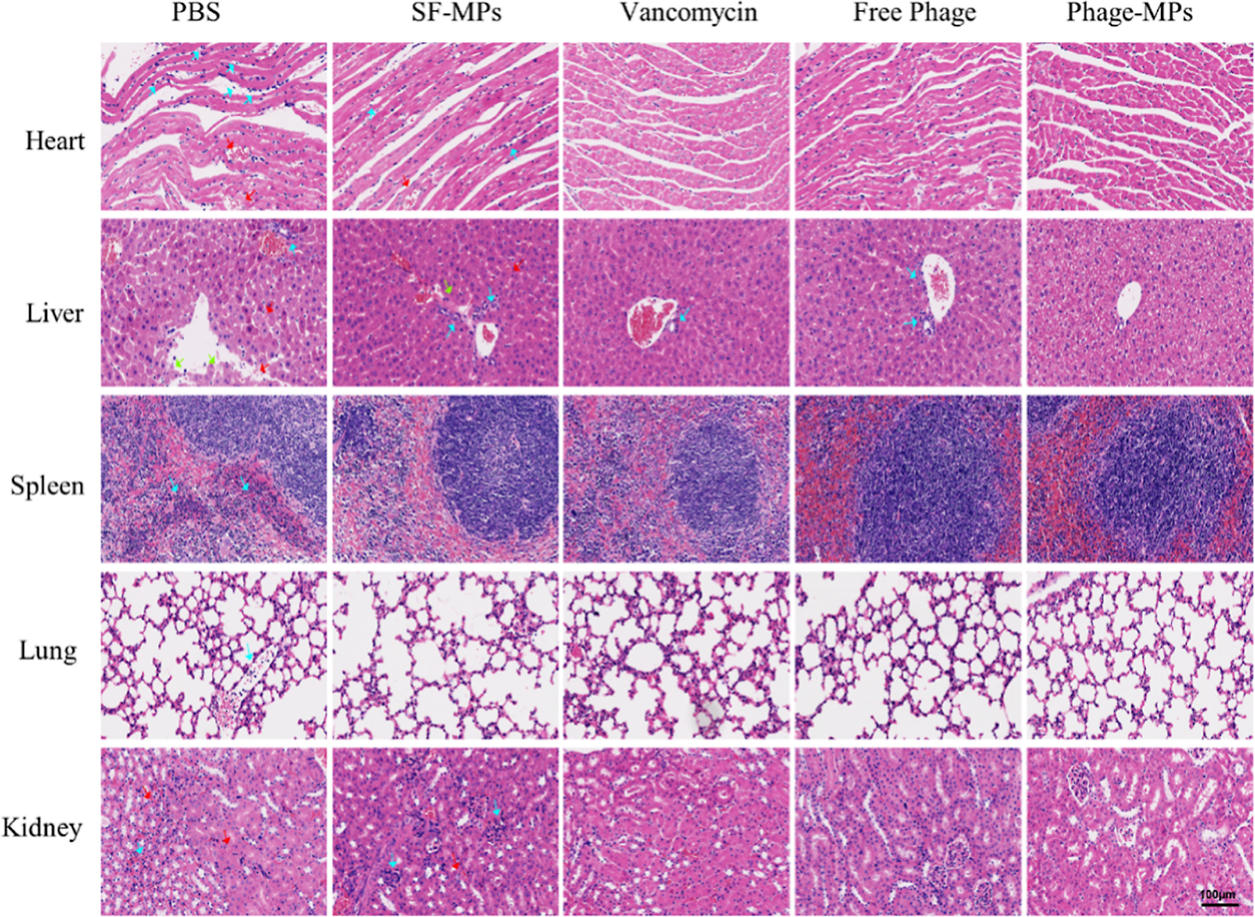
Histopathological H&E staining of the heart, liver, spleen, lung, and kidney from the MRSA-infected mice after different treatments (PBS and SF-MPs at 24 h, Vancomycin, Free Phage, and Phage-MP groups at 72 h). Green arrows mark the immune cells. Red arrows indicate the plasma cells. Green arrows mark the necrosis. The MRSA-infected animals in the PBS and SF-MPs groups died after 24 h, so the figure does not show data from these animals after 24 h.

The in vitro studies have shown the potential of Phage-MPs as a novel therapeutic strategy for bacterial infections, and Phage-MPs had negligible in vitro toxicity, as well. Then, the in vivo effectiveness of Phage-MPs was measured in an abdominal mouse model to evaluate their biomedical application potential. At 0 h, each mouse was intraperitoneally injected with MRSA (OD_600_ = 0.3, 500 μL), as shown in Figure 6A. After 1 h of infection, the mice were treated with 1000 μL of different MPs (1 mg/mL), vancomycin (0.5 mg/mL) as a positive control and PBS as a negative control by injecting them into the peritoneal cavity. The survival rate was 100% for mice treated with vancomycin, Free Phage, and Phage-MPs, while both the PBS group and the SF-MPs group died within 24 h (Figure 6E). Mouse weights initially decreased but showed a subsequent recovery, returning to normal after 3 days (Figure 6D). These results strongly suggest the efficacy of Phage-MPs in treating MRSA-induced infection in vivo and enhancing the overall survival rate in mice. We quantified the bacteria load from the organs of mice infected with bacteria and treated with Phage-MPs at 24, 48, and 72 h. The bacteria number rapidly decreased during observation (Figure 6B, C). At 72 h, almost no bacteria colonies could be found on the plate, indicating that Phage-MPs were effective in eliminating bacteria in vivo. In comparison to the vancomycin and Free Phage groups, the in vivo bactericidal efficiency of Phage-MPs is comparable, substantiating the effective release of phages by SF-MPs for the treatment of bacterial infections in vivo (Figure S6 in the Supporting Information). This underscores the potential of Phage-MPs as an antibiotic replacement strategy, offering an alternative option when antibiotics prove ineffective. Notably, Phage-MPs exhibits advantages over free phages, particularly in terms of storage and transportation. The histological hematoxylin–eosin (H&E) evaluation of the major organ tissues was performed (Figure 7). For the heart, extensive inflammatory infiltration was seen in the PBS group (blue arrow), accompanied by bleeding points (red arrow), and also in the hearts of the SF-MP group. For the liver, apoptosis, some bleeding, and inflammatory cell infiltration were seen in the PBS and SF-MP groups. For the spleen, a significant inflammatory infiltration was observed in the PBS group at 24 h, along with white pulp structure disorder and red pulp hyperplasia. For the lung and kidney, both the PBS and SF-MP groups presented inflammatory infiltration. In contrast, no significant pathological changes were observed in the other three groups. These observations further confirm the biosafety of the Phage-MPs, which showed no significant side effects and did not cause any organ damage while effectively killing bacteria. During bacterial infection, rapid increases in white blood cells, particularly neutrophils, signify active participation in inflammation, contributing to pathogen clearance and tissue repair. Subsequent controlled inflammation results in a gradual return of white blood cell levels to normal, offering insights into the immune status. In Figure S7, the Free Phage group exhibited a higher white blood cell count than the vancomycin and Phage-MP groups, indicating more intense inflammation in the Free Phage group. In comparison to the Vancomycin group, the Phage-MP group reached equilibrium faster, suggesting controlled inflammation and quicker recovery and possibly an anti-inflammatory effect of SF-MPs. Overall, these results demonstrate the effective in vivo bacterial infection treatment of hepatocellular carcinoma by Phage-MPs.

## 4 Conclusions

In summary, we demonstrated the effectiveness of SF-MPs loaded with bacteriophages by simple electrostatic adsorption in killing MRSA. First, Phage-MPs can rapidly release phages in the initial stage to efficiently kill bacteria while also maintaining a slower release in the later stages to treat persistent infections. In vitro experiments showed that the Phage-MPs had a strong antibacterial effect against MRSA, with the bactericidal efficiency reaching 99.9% at 400 μg/mL. Furthermore, the Phage-MPs had desirable biocompatibility and negligible toxicity both in vitro and in vivo. More significantly, excellent therapeutic efficacy was achieved in an abdominal mouse model with no side effects. The ability of Phage-MPs to effectively deliver phages to bacterial infection sites offers significant advantages over conventional antibiotics, such as their specificity to target only the infecting bacteria and their ability to prevent the emergence of drug-resistant strains. It is essential to recognize that clinical infections typically involve complex, multistrain mixtures rather than a single strain. In addressing such scenarios, a phage cocktail therapy approach can be employed. Notably, 95% of the isolated bacteriophages exhibit tail structures and similarly charged surfaces. Consequently, the method outlined in this work is universally applicable. It enables the simultaneous electrostatic adsorption of one or more bacteriophages, offering a versatile response to diverse infections. This approach can potentially address the urgent need for new and effective treatments for bacterial infections, particularly those caused by antibiotic-resistant bacteria.

## Supporting Information Available

Schematic diagram of the preparation of SF microparticles, SEM image of SF-MPs, schematic diagram of isolation and purification of phage specifically infecting *S. aureus* from sewage, plaque morphology of isolated phage specifically infecting *S. aureus*, quantification of phages loaded on MPs, quantitative assessment of bacteria counts of heart, liver, spleen, lung, kidney, and blood from the MRSA-infected mice after different treatments, and changes of inflammatory cell concentrations after different treatments (PDF)
