# Supplementary material for: Injectable Phage-Loaded Microparticles Effectively Release Phages to Kill Methicillin-Resistant Staphylococcus aureus
Source: ACS Appl Mater Interfaces. 2024 Mar 30;16(14):17232–41. doi: 10.1021/acsami.3c19443 (PMC11009905; doi:10.1021/acsami.3c19443)
Supplement: Supplementary file 1 — am3c19443_si_001.pdf [file am3c19443_si_001.pdf]

Supporting Information

Injectable Phage-loaded Microparticles Effectively Release Phages to Kill Methicillin-Resistant *Staphylococcus aureus*

Yajing Xu[1], Tao Yang[1], Yao Miao[1], Qinglei Zhang[2], Mingying Yang[2,*], Chuanbin Mao[3,*]

1. School of Materials Science and Engineering, Zhejiang University, Hangzhou, 310058 Zhejiang, China
2. Institute of Applied Bioresource Research, College of Animal Science, Zhejiang University, Yuhangtang Road 866, Hangzhou, 310058 Zhejiang, China
3. Department of Biomedical Engineering, The Chinese University of Hong Kong, Shatin, Hong Kong SAR, China

E-mail: Chuanbin Mao (cmao@cuhk.edu.hk); Mingying Yang (yangm@zju.edu.cn)

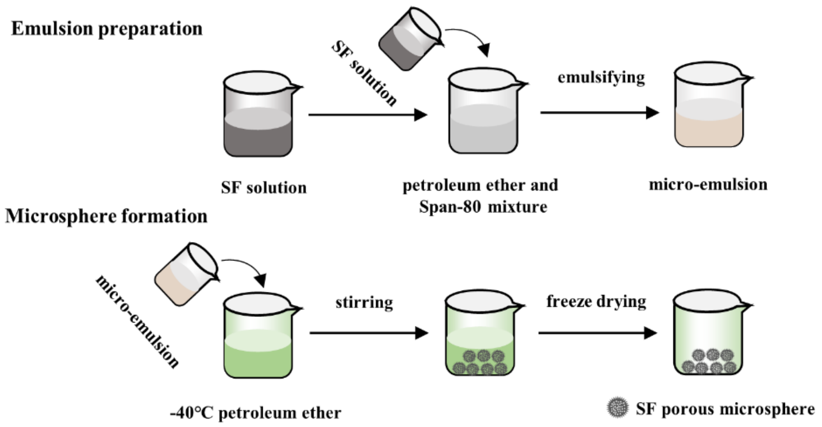

Figure S1. Schematic diagram of the preparation of SF microparticles.

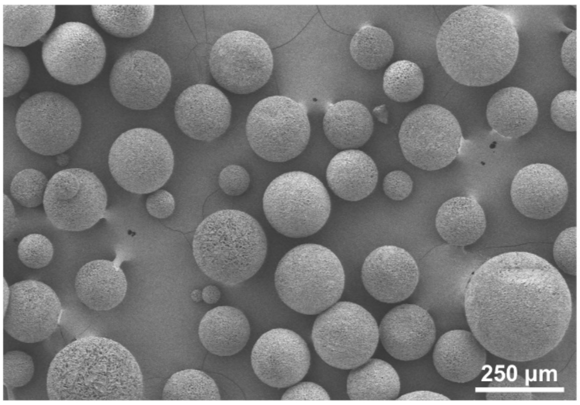

Figure S2. SEM image of SF-MPs.

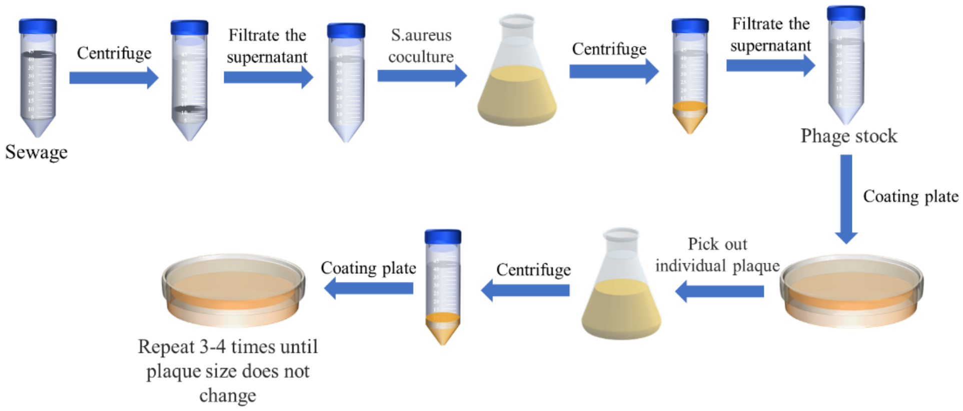

Figure S3. Schematic diagram of isolation and purification of phage specifically infecting S. aureus from sewage.

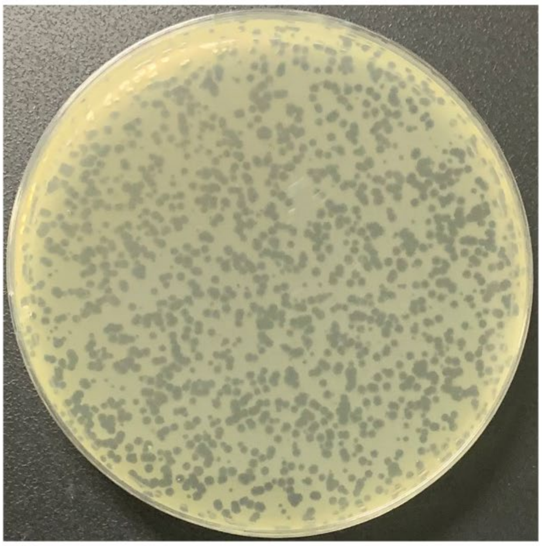

Figure S4. Plaque morphology of isolated phage specifically infecting S. aureus.

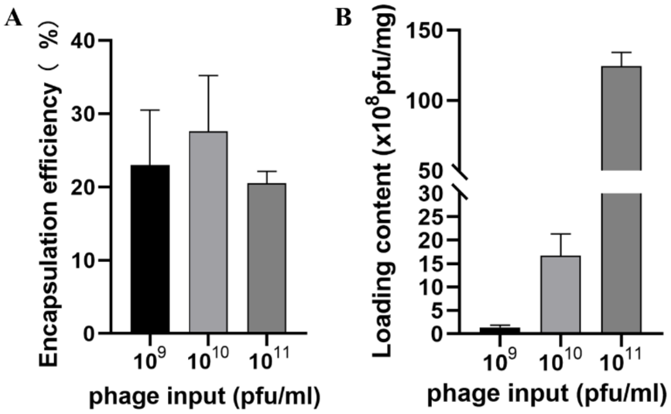

Figure S5. Quantification of phages loaded on MPs. The phage encapsulation efficiency (A) and loading content (B) of different phage inputs electrostatically adsorbed in SF-MPs.

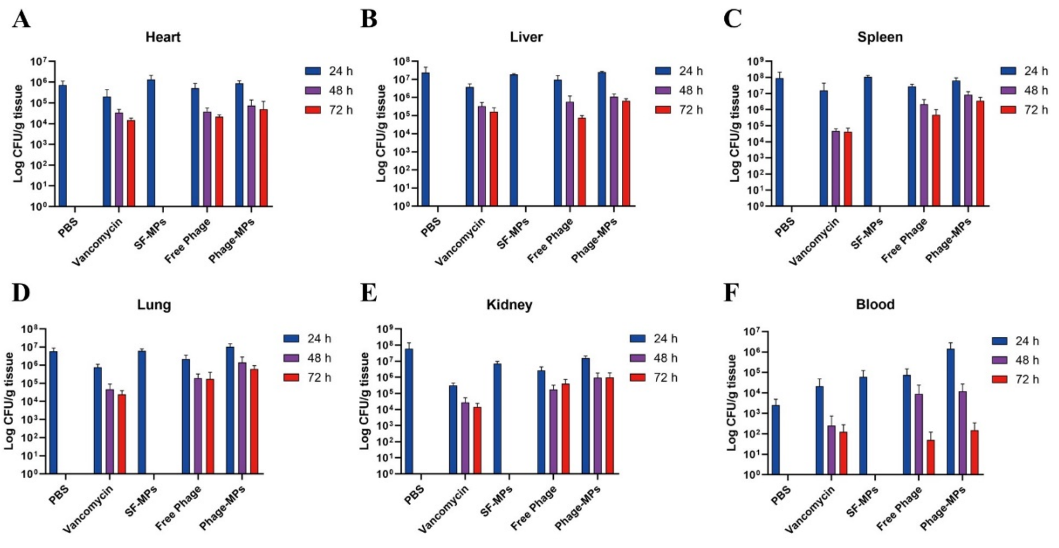

Figure S6. Quantitative assessment of bacteria counts of heart (A), liver (B), spleen (C), lung (D), kidney (E) and blood (F) from the MRSA-infected mice after different treatments. The MRSA-infected animals in the PBS and SF-MPs groups died after 24 h, so the figure does not show data from these animals after 24 h.

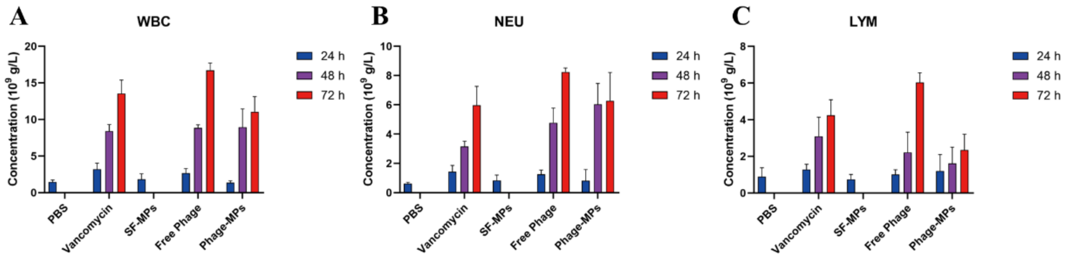

Figure S7. Changes of inflammatory cell concentrations after different treatments. (A) White blood cells. (B) Neutrophile granulocytes. (C) Lymphocytes.
